# An Electrochemical Study on the Copolymer Formed from Piperazine and Aniline Monomers

**DOI:** 10.3390/ma11061012

**Published:** 2018-06-14

**Authors:** Samiha Dkhili, Sara López-Bernabeu, Chahineze Nawel Kedir, Francisco Huerta, Francisco Montilla, Salma Besbes-Hentati, Emilia Morallon

**Affiliations:** 1 Laboratoire de Chimie des Matériaux, Faculté des Sciences de Bizerte, Zarzouna Université de Carthage, Jarzouna, Bizerte 7021, Tunisia; samihadkhili@yahoo.fr (S.D.); salma.hentati@fsb.rnu.tn (S.B.-H.); 2 Departamento de Química Física e Instituto Universitario de Materiales, Universidad de Alicante, Ap. 99, E-03080 Alicante, Spain; sara.lopez@ua.es (S.L.-B.); kedir.nawel@hotmail.fr (C.N.K.); francisco.montilla@ua.es (F.M.); 3 Departamento de Ingenieria Textil y Papelera, Universitat Politecnica de Valencia, Plaza Ferrandiz y Carbonell, 1, E-03801 Alcoy, Spain; frahuear@txp.upv.es

**Keywords:** copolymer, polyaniline, piperazine, FTIR in situ

## Abstract

A study on the electrochemical oxidation of piperazine and its electrochemical copolymerization with aniline in acidic medium is presented. It was found that the homopolymerization of piperazine cannot be achieved under electrochemical conditions. A combination of electrochemistry, in situ Fourier transform infrared (FTIR), and ex situ X-ray photoelectron spectroscopy (XPS) spectroscopies was used to characterize both the chemical structure and the redox behavior of an electrochemically synthesized piperazine–aniline copolymer. The electrochemical sensing properties of the deposited material were also tested against ascorbic acid and dopamine as redox probes.

## 1. Introduction

Electrochemical devices based on conducting polymers, either working as sensors or as systems taking advantage of other electrocatalytic effects, have become a topic of growing interest for molecular electrochemistry during the last decades [1,2,3,4,5,6]. It is known that conducting polymers show the ability to incorporate catalytic molecules, and numerous works based on this particular property led to interesting applications in the field of bioelectrochemical sensing. The polymer constitutes an organic matrix where catalytic molecules, such as enzymes, may preserve its activity better, and where the conducting surroundings may electrically wire it to the metal electrode surface [7,8,9,10].

Besides the incorporation of catalytic species, the pristine conducting polymers (polyaniline, polypyrrole, etc.) can be also chemically modified to gain further catalytic capabilities. The most classical way to perform chemical modification is to copolymerize aniline or pyrrole, for example, with monomers that are able to provide the final material with the desired catalytic features. In this context, chemical derivatives of piperazine (diethylenediamine) constitute a promising group of catalytic molecules that have been successfully applied in chemical and electrochemical sensing [6,11,12,13,14]. In spite of this, few studies exist that are devoted to the exploration of the catalytic properties of polymer systems containing the parent piperazine molecule. Among them, it has been reported that a novel piperazine-functionalized mesoporous organic polymer exhibited highly catalytic activity and selectivity for some organic synthesis reactions in aqueous medium [15]. The electrochemical sensing ability of piperazine in combination with inorganic polymers has been explored recently in the selective detection of ascorbic acid [16]. The sensing system was a piperazine-functionalized mesoporous silica, and the results show that this type of hybrid material is a potential candidate for the construction of bioelectrochemical sensors. 

**Scheme 1:**
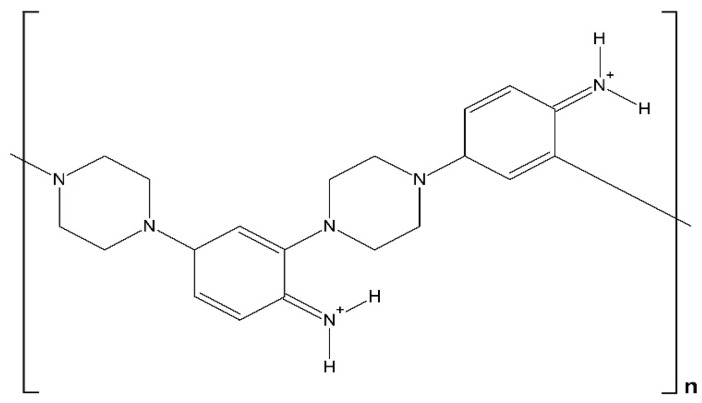
Model structure proposed by Kabilan et al. [17] for the chemically synthesized piperazine–aniline copolymer in the doped state.

The chemical copolymerization of aniline and piperazine was studied by Ramachandran et al. [17]. Although the catalytic properties of the obtained material were not analyzed, the copolymer showed electrochemical activity. The chemical structure proposed (see Scheme 1) seems constituted by alternated piperazine and aniline moieties, which are bound through aniline *ortho*- and *para*-positions. It was shown that charge delocalization in this polymer includes also oxidized piperazine centers, but extended conjugation was not observed. The electrical conductivity of the material was in the range of 10^−7^–10^−9^ S cm^−1^. 

The goal of the present contribution is to study the electrochemical oxidation of piperazine in acidic medium and, additionally, its electrochemical copolymerization with aniline. A combination of in situ Fourier transform infrared (FTIR) spectroscopy and electrochemistry will be used to characterize the redox behavior of the copolymer, while X-ray photoelectron spectroscopy (XPS) will shed more light on the chemical structure of the electrochemically synthesized material in comparison with the chemically obtained one. Finally, the electrochemical sensing properties of the copolymer will be tested against ascorbic acid and dopamine.

## 2. Experimental

The background electrolyte employed for the studies was perchloric acid (Merck Suprapur, Merck Group, Darmstadt, Germany), and the solutions were prepared with 18.2 MΩ cm water obtained from an Elga Labwater Purelab (Elga-Veolia, High Wycombe, UK) system. Piperazine (98.8%), aniline (99.5%), ascorbic acid (AA, 98.9%), and dopamine (DA, 97.9%) were purchased from Merck. Cyclic voltammetry experiments were carried out in a conventional three-electrode cell under N_2_ atmosphere. The working electrode was a polycrystalline platinum sphere, and a platinum wire was used as the counter electrode. All of the potentials were measured against the reversible hydrogen electrode (RHE) immersed in the same electrolyte through a Luggin capillary. Cyclic voltammograms were recorded at a constant sweep rate of 0.05 V s^−1^ and at room temperature. The platinum electrodes were thermally cleaned and subsequently protected from the laboratory atmosphere by a droplet of ultrapure water. 

A Nicolet 5700 spectrometer (Thermo Electron Scientific Instruments, Madison, WI, USA) equipped with an N_2_-cooled mercury cadmium telluride detector was employed for the in situ FTIR experiments. The working Pt disc electrode was mirror-polished with alumina powder, and the spectroelectrochemical cell used a prismatic CaF_2_ window beveled at 60° in order to increase the beam intensity reaching the infrared (IR) detector. All of the spectra were collected at the same 8 cm^−1^ resolution using deuterated water (99.9% D) as the solvent. The processed spectra have been presented in the standard mode ΔR/R.

A VG-Microtech Multilab 3000 electron spectrometer (VG Microtech Ltd., Uckfield, UK) was employed to acquire the ex situ XPS spectra. The 300-W power radiation source was a non-monochromatized Mg-Kα, and the analysis was performed under 5 × 10^−7^ Pa pressure. The high-resolution spectra were acquired at 50-eV pass energy, and are presented as a combination of Lorentz (30%) and Gaussian (70%) curves. The C 1s line at 284.4 eV has been employed as the reference for the experimental binding energies, which were obtained with 0.2 eV accuracy.

The scanning electron micrographs were acquired by means of an ORIUS-SC600 Field Emission Scanning Electron Microscope (FE-SEM) (Gatan Inc., Pleasanton, CA, USA), which was equipped with a ZEISS microscope (Carl Zeiss Microscopy Ltd., Cambridge, UK).

## 3. Results and Discussion

### 3.1. Electrochemical Behavior of Piperazine on Pt

**Figure 1:**
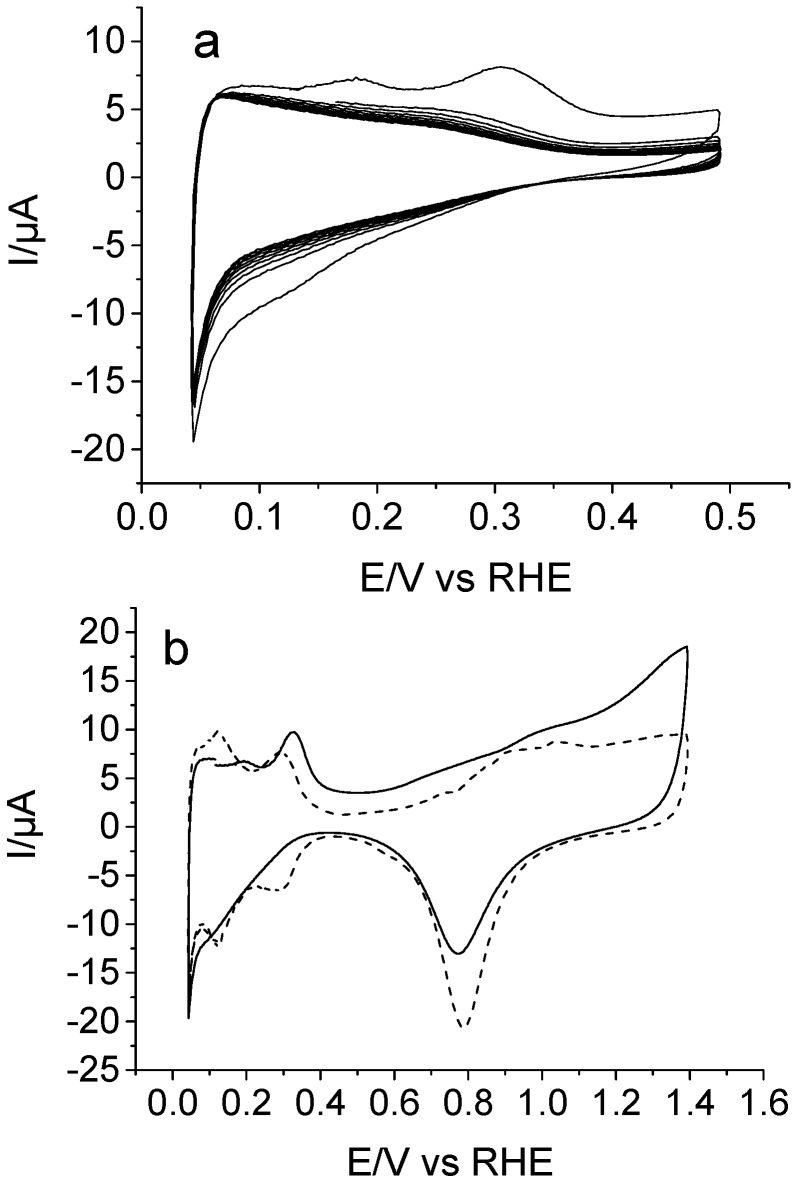
Cyclic voltammograms recorded for a Pt electrode in 1 M of HClO_4_ solution containing 10 mM of piperazine. (**a**) Electrochemical behavior within the potential region 0.05–0.5 V (10 cycles); (**b**) Electrochemical response obtained during the first excursion of up to 1.4 V (solid line), and for clean Pt, in 1 M of HClO_4_ free of piperazine (dashed line) in the same potential window. v = 50 mV s^−1^ in all cases.

Cyclic voltammetry (CV) curves recorded for a polycrystalline platinum electrode immersed in 1 M of HClO_4_ + 10 mM piperazine solution are illustrated in Figure 1. The electrode was immersed at a controlled potential of 0.1 V, and the response was firstly examined in the 0.05–0.5 V potential range (Figure 1a). Two oxidation peaks were observed at 0.18 and 0.30 V during the forward scan up to 0.5 V. Both features are related with the electrochemistry of partially blocked adsorption sites at the platinum surface in perchloric medium [18], which reveals that piperazine strongly adsorbs on this electrode. In a new experiment, the clean electrode was immersed at 0.1 V, and the potential was scanned up to 1.4 V to examine the anodic behavior of piperazine. The onset of oxidation occurs at about 0.5 V, but the electrochemical process appears more clear at potentials higher than, roughly, 1.0 V in the form of a broad current, with no well-defined peaks. The voltammetric profile recorded during the reverse scan does not reach the characteristic shape of a Pt electrode (Figure 1b, dashed line). This result indicates that some adsorbed species coming from piperazine oxidation still remain on the electrode surface and block part of Pt adsorption sites. However, as expected, no electropolymerization of piperazine is observed after continuous potential cycling, with the voltammetric profile being almost equivalent to that shown in the solid line of Figure 1b. 

**Figure 2:**
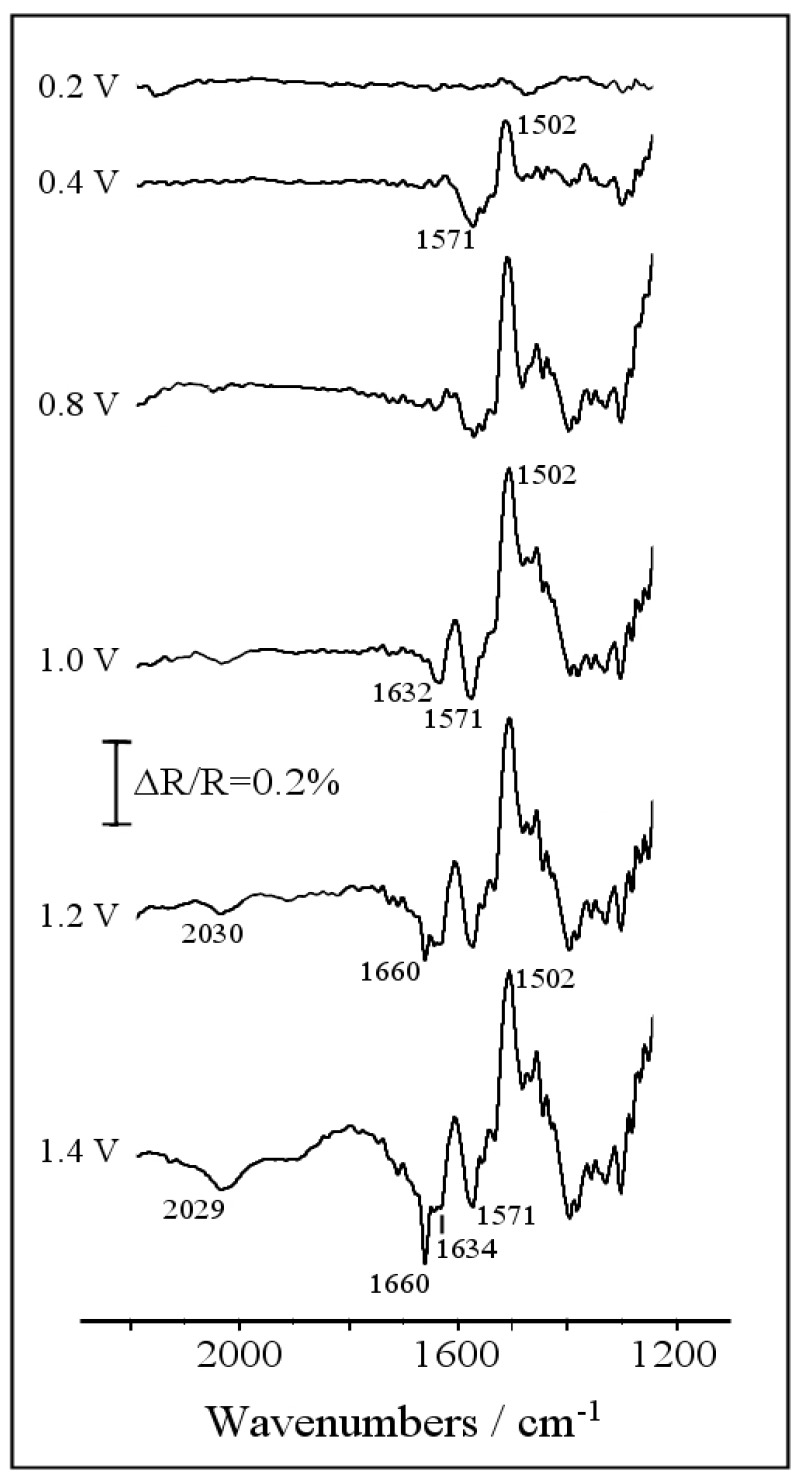
Set of in situ Fourier transform infrared (FTIR) spectra collected during the oxidation of 10 mM of piperazine in 0.1 M of HClO_4_/D_2_O solution. Reference potential: 0.1 V. Sample potential labeled for each spectrum: 100 interferograms at each potential.

In situ FTIR spectroscopy has been used to increase the understanding of the piperazine oxidation process. Figure 2 shows a set of spectra obtained for a Pt electrode immersed in 10 mM of piperazine + 0.1 M HClO_4_, using D_2_O as the solvent_._ The mirror-polished platinum electrode was transferred to the spectroelectrochemical cell, which was immersed at 0.1 V into the solution, and its surface was pressed against the CaF_2_ window. In this case, the concentration of perchloric acid was 0.1 M in order to avoid the damage of the spectroscopic window. The reference spectrum was collected at 0.1 V, and then, the potential was stepped up to 1.4 V to collect several sample spectra. By referring each sample to the unique reference, the information on the redox transformations undergone by the piperazine as a function of the applied potential can be obtained. A positive-going absorption feature appears at 1502 cm^−1^ in the spectrum obtained at 0.4 V, whose intensity rises significantly at higher applied potentials. This means that the species giving rise to this vibrational mode disappears upon oxidation. The frequency of 1502 cm^−1^ is compatible with the –ND_2_^+^ stretching vibration of deuterated piperazine [19], which occurs because of the proton–deuterium exchange equilibrium in D_2_O solvent. The electrochemical oxidation of piperazine at higher potential values results in the formation of different carbonyl groups within the piperazine ring, as deduced from the C=O stretching vibrations appearing at around 1570 and 1630 cm^−1^ [20,21].

Finally, the spectra collected at large anodic potentials display two additional negative bands at 1660 and 2030 cm^−1^. The former seems related with the occurrence of C=O in amide species, while the frequency of the latter strongly suggests the formation of multiple C-N bonds, probably as isocyanates [22]. It is known that piperazine N-oxides obtained from the oxidation of piperazine show N-O stretching frequencies at around 1350 cm^−1^ [23]. The presence of this kind of structure cannot be ruled out during the electrochemical oxidation, because the frequency region between 1250 and 1450 cm^−1^ is altered in the spectra of Figure 2 due to the presence of diverse C-N, CH_2_, CND, and N-D absorptions. Anyway, from the results presented in this section, it is derived that the electrochemical oxidation of piperazine on Pt electrodes yields some kind of ketopiperazine species at moderate potentials and, at more positive potential values, the ring could open to produce both amide groups and isocyanates.

### 3.2. Electrochemical Copolymerization of Piperazine and Aniline

**Figure 3:**
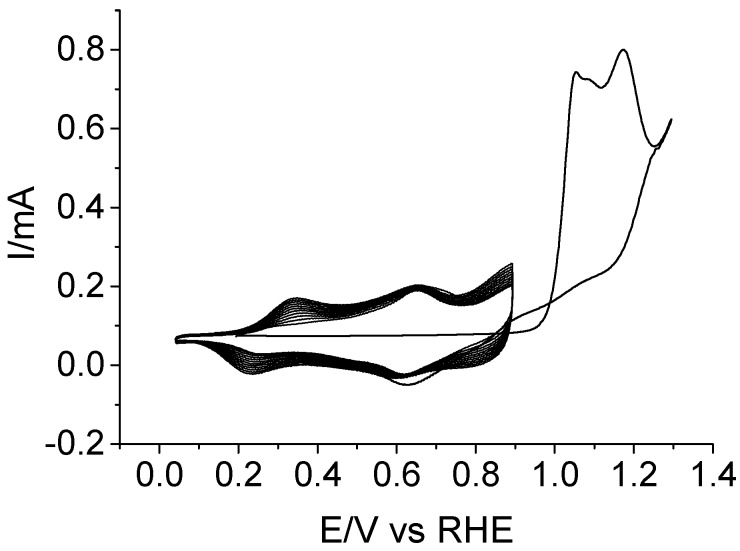
Cyclic voltammograms recorded for a Pt electrode during the electrochemical copolymerization of 0.5 M of piperazine and 0.1 M of aniline in 1 M of HClO_4_ solution. The upper potential limit was set at 1.3 V for the first scan, and at 0.9 V for the subsequent ones. v = 50 mV s^−1^.

The results in the previous section strongly suggest that the copolymerization of piperazine with aniline should be carried out using as low a potential as possible, in order to minimize the irreversible oxidation of the former. However, it is known that anilinium cations (which originate at potentials beyond 1.2 V versus RHE) are needed to trigger the deposition of polyaniline-derived polymers. A compromise is then needed between the most favorable polymerization conditions to obtain a little degraded material, and the actual conditions to obtain a deposit. Figure 3 shows the experiment carried out to achieve electropolymerization under the established premises. Owing to the higher reactivity of aniline monomer, the copolymerization solution contained a piperazine:aniline relative concentration as large as 5:1 in 1 M of HClO_4_. The first potential scan was carried out up to 1.3 V to generate an adequate amount of anilinium radicals, while the inversion potential was set at 0.9 V for the subsequent scans to ensure that the piperazine unbroken rings can be incorporated to the growing polymer. The development of new redox processes within the 0.05–0.9 V potential region evidences the growth of an electroactive polymeric species. After 10 potential cycles, the Pt electrode was removed from the solution, and its surface appeared covered by a dark blue film. 

**Figure 4:**
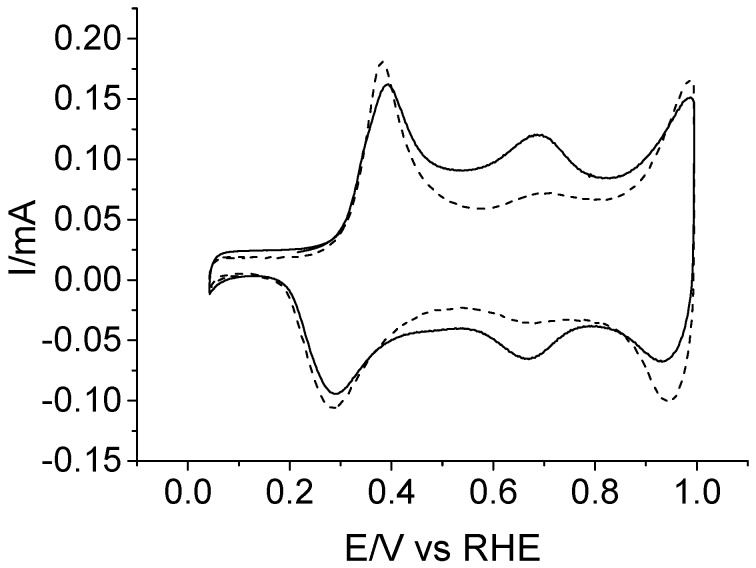
Electrochemical response in 1 M of HClO_4_ medium of a Pt electrode covered with either polyaniline (dashed line) or an aniline–piperazine copolymer (solid line), which were deposited under the potential program used in Figure 3. v = 50 mV s^−1.^

The electrochemical behavior of the deposited copolymer was tested in an acidic background solution that was free of any monomer species, and the result is shown in Figure 4 (solid line). CV shows three redox transitions centered at around 0.33, 0.68, and 0.97 V. The first one can be assigned to a leucoemeraldine–emeraldine transformation similar to that of pristine polyaniline. The second one, which is broader and less intense, has been usually interpreted in terms of the presence of different quinoid structures [24,25]. For the copolymer studied here, the formation of ketopiperazines upon piperazine oxidation at very low anodic potentials (see Figure 2) demonstrates that the deposited material could incorporate a little amount of those previously formed quinoid structures. However, the high relative intensity of the voltammetric wave at 0.68 V strongly suggests a main contribution of active redox centers involving piperazine units which are oxidized *after* they are incorporated to the copolymer chain. Accordingly, the second redox peak may be related to the existence of redox transitions involving hydroxypiperazine ⇌ ketopiperazine species within the copolymer structure [26]. With regard to the pair of redox peaks centered at 0.97 V in the CV of Figure 4, they can be clearly related to the emeraldine–pernigraniline transition of the copolymer, which is similar to that undergone by polyaniline under the same experimental conditions (dashed line).

**Figure 5:**
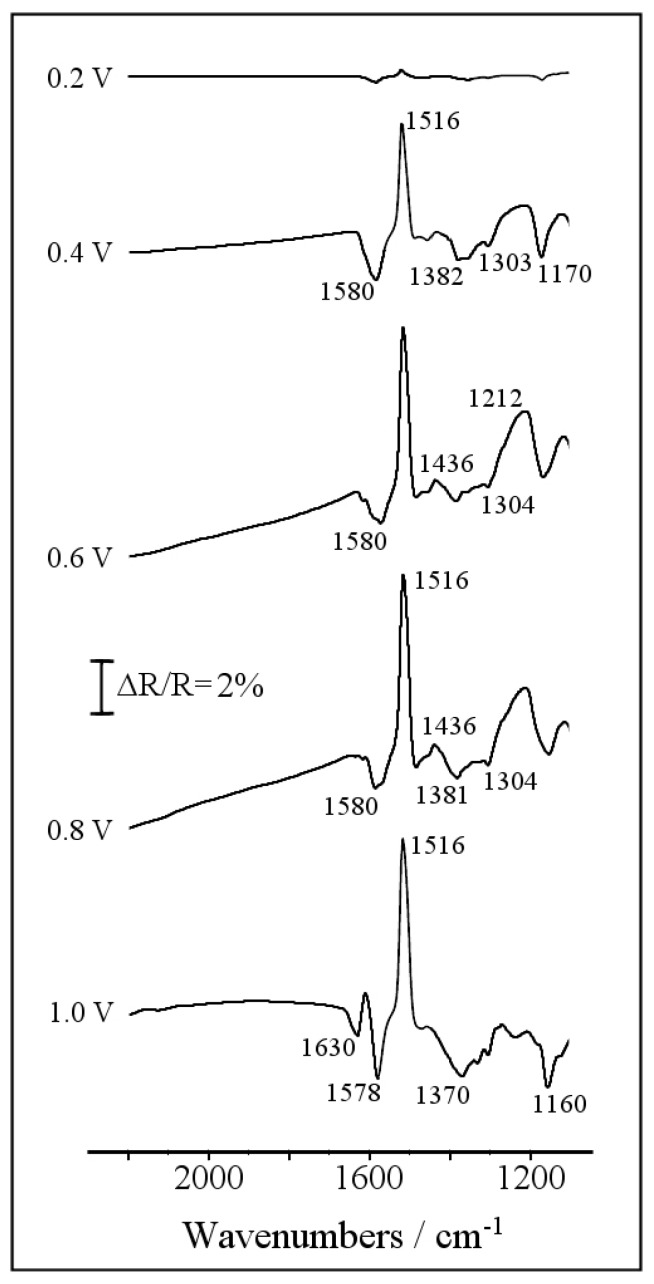
Set of in situ FTIR spectra collected during the oxidation of an electrochemically obtained poly(aniline-co-piperazine) film in 0.1 M of HClO_4_/D_2_O test solution. Reference potential 0.1 V. Sample potential labeled for each spectrum: 100 interferograms at each potential.

In order to monitor the redox behavior of the copolymer and analyze the chemical nature of the species involved in the redox transitions (particularly the existence of a keto-hydroxypiperazine transformation), in situ FTIR spectroscopy experiments were performed for the copolymer. The Pt-modified electrode was transferred to the IR spectroelectrochemical cell, which contained a perchloric acid solution that was free of monomers and prepared with D_2_O to facilitate assignments in the 1500–1700 cm^−1^ spectral range. After some potential cycles within the stability window of the copolymer, the Pt surface was pressed against the prismatic CaF_2_ window, and a reference spectrum was collected at 0.1 V. Finally, the potential was stepped to higher values to collect sample spectra, and the results are displayed in Figure 5. Three main positive bands at 1516, 1436, and 1212 cm^−1^, and three clear negative bands at 1630, 1580, and 1170 cm^−1^ can be observed together with several features in the 1300–1400 cm^−1^ region. Some of the referred absorptions can be unambiguously assigned to the presence of a polyaniline skeleton. Particularly, the complete disappearance of the leucoemeraldine state at 0.6 V is evidenced by the vanishing of the aromatic C-C stretching mode at 1516 cm^−1^ and of the C-N-C stretching at 1212 cm^−1^ [27,28]. The formation of oxidized emeraldine (0.6 V) and pernigraniline (1.0 V) structures is also supported by the development of quinoid C=C stretching vibrations at 1580 cm^−1^ [28,29], by the -CH in-plane bending at oxidized aniline rings at 1170 cm^−1^, and, finally, by the generation of several intermediate-order C-N vibrations in the 1300–1400 cm^−1^ frequency window [29,30]. On the other hand, the successful incorporation of piperazine structures to the polyaniline chain is evidenced by two representative bands, which cannot be observed for a pristine polyaniline. These absorptions correspond to the activation of the CH_2_ bending upon oxidation (positive-going feature at 1435 cm^−1^ [31]), and to the carbonyl C=O stretching at 1630 cm^−1^. This latter band supports the voltammetric result in Figure 3, and confirms that a significant fraction of piperazine rings are present in the form of electroactive ketopiperazines (C=O ⇌ C-OH). The absence of additional vibrations at around 1660 and 2030 cm^−1^ shows that neither amide structures nor isocyanates are formed and, consequently, that piperazine was not overoxidized during the electropolymerization process under the experimental conditions employed. FTIR assignments are collected in the Supplementary Material (Table S1).

**Figure 6:**
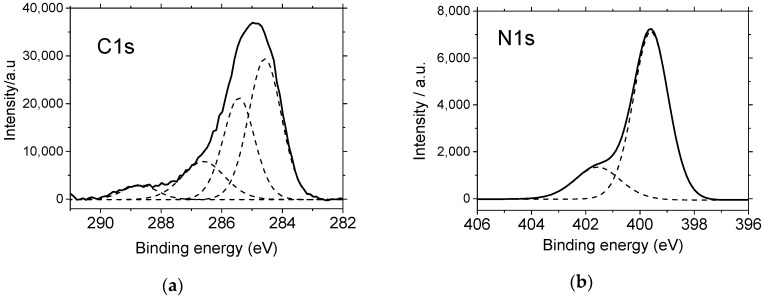
Deconvoluted (**a**) C 1s and (**b**) N 1s X-ray photoelectron spectroscopy (XPS) core-level spectra of an electrodeposited aniline–piperazine copolymer. The sample was obtained as in Figure 3.

Additionally, the electrodeposited copolymer was examined by ex situ XPS in order to analyze its surface composition, and also to give support to the chemical structures suggested by in situ FTIR spectroscopy. A film grown after 10 voltammetric cycles as in Figure 3 was rinsed with ultrapure water, dried under nitrogen, stored in a dry place for 24 h, and then analyzed by XPS. Figure 6 shows the photoelectronic spectra of C 1s and N 1s core levels. The C 1s signal can be fitted with four peaks at 284.5, 285.4, 286.6, and 288.7 eV. Both the high energy level and the weak intensity of the latter contribution are compatible with the presence of a small amount of carbonyl carbon, which was probably associated to the ketopiperazine centers. On the other hand, there are two major signals that were undoubtedly associated to aromatic carbon, and hence to aniline rings. This is the main peak at 284.6 eV, which was attributed to plain aromatic carbon, and the signal at 285.4 eV, which was due to aromatic carbon bonded to neutral nitrogen. On the other hand, binding energies at around 286.6 eV are characteristic of carbon bonded to positive nitrogen [32], and consequently, this peak is compatible with the presence of piperazine rings within the polymer backbone. The best fit for the N 1s spectrum shows only two contributions at 399.6 and 401.6 eV, but unfortunately, it is not possible to distinguish signals coming from the piperazine and aniline environments. The peak at 399.6 eV is clearly attributed to neutral nitrogen, but it could be associated to any of the amine, imine or even amide groups, as these species do not show significantly different chemical shifts. In the same way, the higher binding energy signal at 401.6 eV is compatible with the presence of piperazine within the material. That peak is assigned to positively charged nitrogen atoms resulting from the protonation of imine centers (located exclusively at aniline rings) and secondary amine positions (at both piperazine and aniline rings) [32].

**Figure 7:**
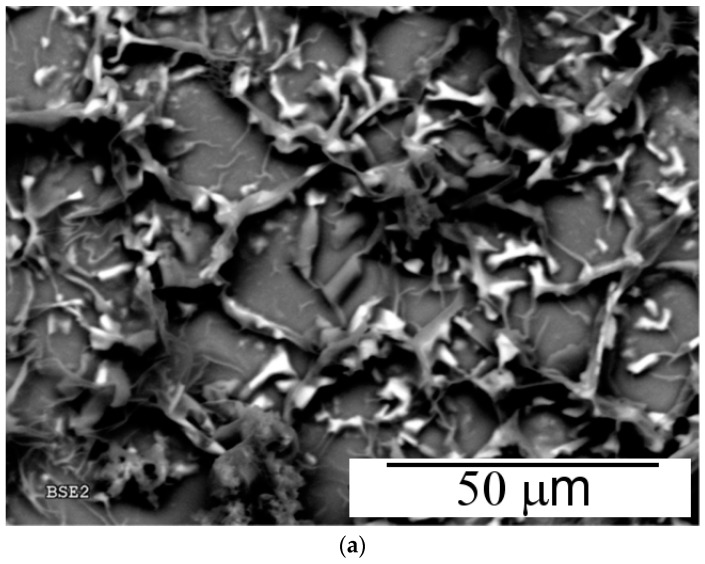

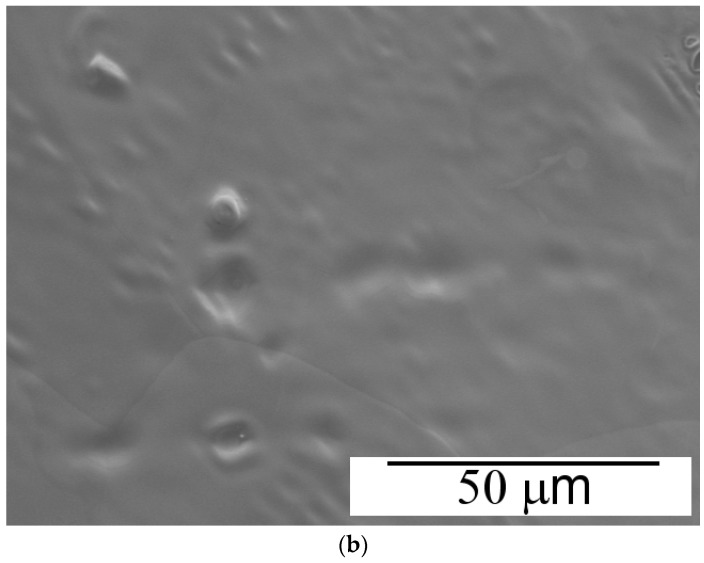
Scanning electron micrographs at 1000× magnification of copolymer (**a**) and polyaniline (**b**) deposited under the same experimental conditions.

Chemically obtained polyanilines are usually amorphous solids, but more or less ordered structures can also be obtained depending on the synthesis conditions [33]. In general, better ordering is observed for electrochemically-prepared thin films [34]. The surface morphology of the aniline–piperazine copolymer electrodeposited on platinum after 10 cycles has been examined by SEM. The top image in Figure 7 shows how this organic coating is completely distributed over the surface in the form of flat ribbon strings with a width of about 3–5 μm. These structures are quite different from those detected for unmodified polyaniline deposited on Pt under similar experimental conditions, for which a uniformly distributed, smooth film is obtained (Figure 7b). According to these observations, the presence of a significant amount of piperazine units is seen at the origin of the particular morphologic features shown by the copolymer material.

### 3.3. Testing the Electrocatalytic Properties of the Copolymer towards Dopamine and Ascorbic Acid

**Figure 8:**
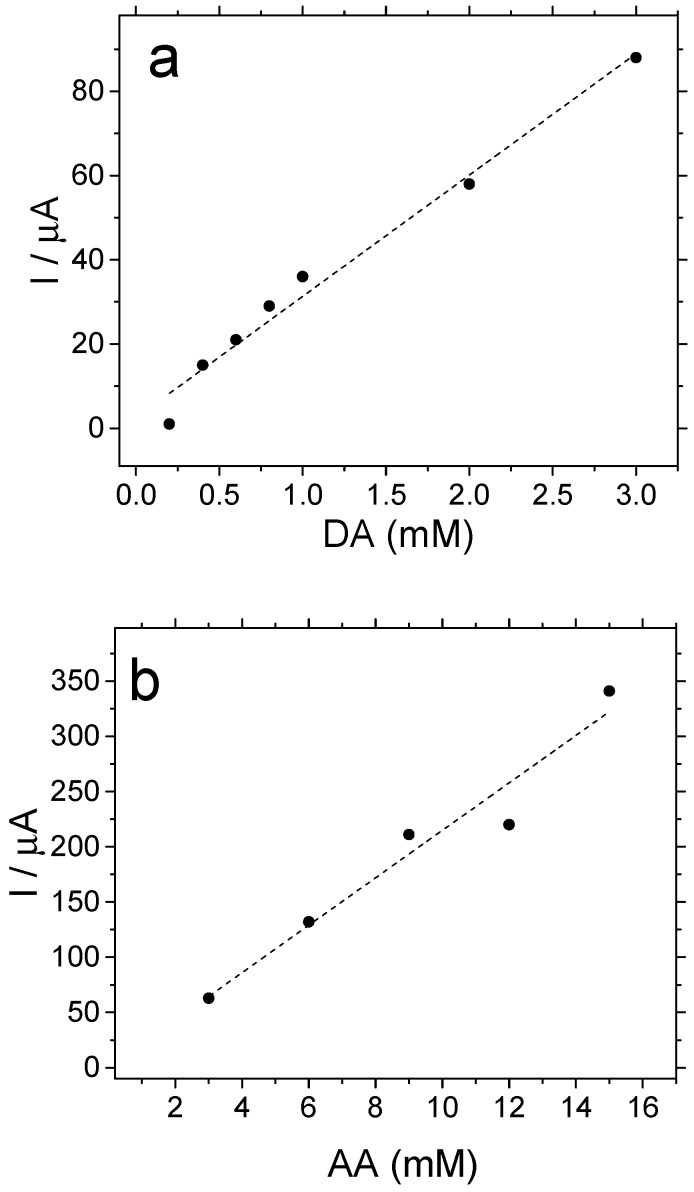
Peak oxidation currents recorded for a Pt electrode covered with a piperazine–aniline copolymer in 1 M of HClO_4_ aqueous solutions containing an increasing concentration of analyte: (**a**) dopamine; (**b**) ascorbic acid.

Platinum electrodes coated with copolymer films have been employed to determine dopamine (DA) and ascorbic acid (AA) in synthetic samples. The sensitivity of the measurement and the catalytic performance of the copolymer have been evaluated in acidic medium. First, DA samples were prepared within a concentration range from 0.2 to 3.0 mM. Then, the oxidation current of this analyte was recorded for the Pt electrode covered with the copolymer at a potential of 0.85 V, which corresponds to the first anodic peak of the DA→DQ reaction (see Figure S1 in Supplementary Materials for a CV curve). This anodic peak potential is nearly the same as that reported in the literature for bare Pt electrodes in acidic medium [35,36]. Figure 8a shows how the oxidation current of dopamine increases almost linearly at increasing analyte concentrations. From that plot, it can be derived that the copolymer demonstrates quite a sensitive response, in the order of 29 μA mM^−1^, within the range of concentrations studied. A similar electrocatalytic effect can be observed in Figure 8b for AA electrooxidation. For such a reaction, cyclic voltammograms recorded with a Pt electrode covered with the copolymer material (see Figure S2 in Supplementary Materials for a CV curve) shows the anodic peak centered at 0.89 V, which is a value slightly below that usually obtained for bare Pt surfaces under similar experimental conditions [37,38]. Also in this case, the determination of AA on the copolymer substrate shows linearly increasing faradaic responses with a sensitivity of about 22 μA mM^−1^.

## 4. Conclusions

The electrochemical oxidation of piperazine on platinum electrodes at moderate potentials (roughly below 1.0 V/RHE) preserves the ring structures and produces ketopiperazines as the main reaction product. In situ FTIR spectroscopy strongly suggested that ring opening and overoxidation occur at higher potentials to form both amides and isocyanates. As a result, it was observed that the homopolymerization of piperazine cannot be achieved in perchloric acid aqueous solution under electrochemical conditions. 

On the contrary, piperazine can be successfully copolymerized with aniline in acidic medium. The deposited copolymer shows some electrochemical features similar to those of pristine polyaniline, particularly those related with leucoemeraldine-to-emeraldine and emeraldine-to-pernigraniline transitions. However, a key difference arises in the intermediate potential region between both transitions. As shown by in situ FTIR and XPS spectroscopies, the intermediate redox peak is a consequence of the incorporation of piperazine units to the copolymer structure. Most of these piperazine centers undergo electrochemical oxidation during the copolymerization potential scans and, as a result, a new reversible hydroxy ⇌ ketopiperazine redox transformation seems to occur as the intermediate voltammetric feature centered at 0.68 V. It should be noted that, owing to the conservative potential program applied during the deposition process, any significant amount of overoxidation products was not incorporated to the copolymer structure. As a result, the deposited material is chemically stable, and presents a well-defined electrochemical behavior.

It was observed that the aniline–piperazine copolymer shows a linear response when applied to the electrochemical determination of dopamine or ascorbic acid in synthetic samples. The sensitivity of the measurement is in both cases high enough to assure the correct quantification of analytes. This electrode material has to be tested in real samples, but according to the results presented in this contribution, it shows potential application in DA and AA sensors, owing to its facile synthesis, high chemical stability, and reproducible linear response.

## Supplementary Materials

The following are available online at http://www.mdpi.com/1996-1944/11/6/1012/s1, Figure S1: Linear Sweep Voltammogram showing the oxidation of 3 mM DA on a Pt electrode covered with the aniline-piperazine copolymer. DA oxidation peak is centered at 0.85 V, Figure S2: Linear Sweep Voltammogram showing the oxidation of 30 mM AA on a Pt electrode covered with the aniline-piperazine copolymer. AA oxidation peak is centered at 0.89 V, Table S1: Observed frequencies and proposed assignments for the vibrational bands derived from Figure 2 and Figure 5.
