# Supplementary material for: An Electrochemical Study on the Copolymer Formed from Piperazine and Aniline Monomers
Source: Materials (Basel). 2018 Jun 14;11(6):1012. doi: 10.3390/ma11061012 (PMC6025326; doi:10.3390/ma11061012)
Supplement: Supplementary file 1 [file materials-11-01012-s001.pdf]

Supplementary Materials

An Electrochemical Study on the Copolymer Formed from Piperazine and Aniline Monomers

S. Dkhili[1,2], S. López-Bernabeu[2], C.N. Kedir[2], F. Huerta[3], F. Montilla[2], S. Besbes-Hentati[1], E. Morallón[2]

Table S1. Observed frequencies and proposed assignments for the vibrational bands derived from Figure 2 and Figure 5.

	**Frequency / cm⁻¹**		**Suggested Assignment**
	**Piperazine (Figure 2)**	**Copolymer (Figure 5)**	
**Reduced**	1502		$–ND_2^+$ str.
		1212	C-N-C str.
		1436	CH_2 bend.
		1516	C–C str. Aromatic rings
**Oxidized**	1570-1630	1630	C=O str. Carbonyl
		1170	-CH bend.
		1580	C=C str. Quinoid rings
	1660		C=O str. Amide
	1250-1450	1300-1400	Overlapped bands: C-N (intermediate order) -CND-, N-D, Possible N-O in oxidized piperazine
	2030		C≡N str. Isocyanate

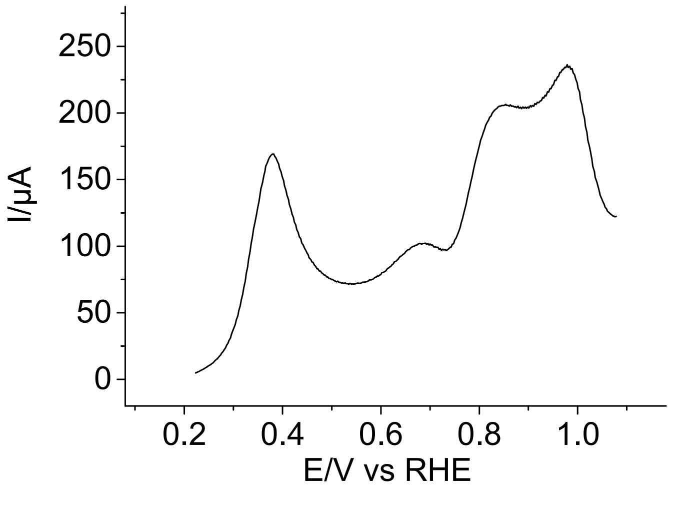

Figure S1. Linear Sweep Voltammogram showing the oxidation of 3 mM DA on a Pt electrode covered with the aniline-piperazine copolymer. DA oxidation peak is centered at 0.85 V.

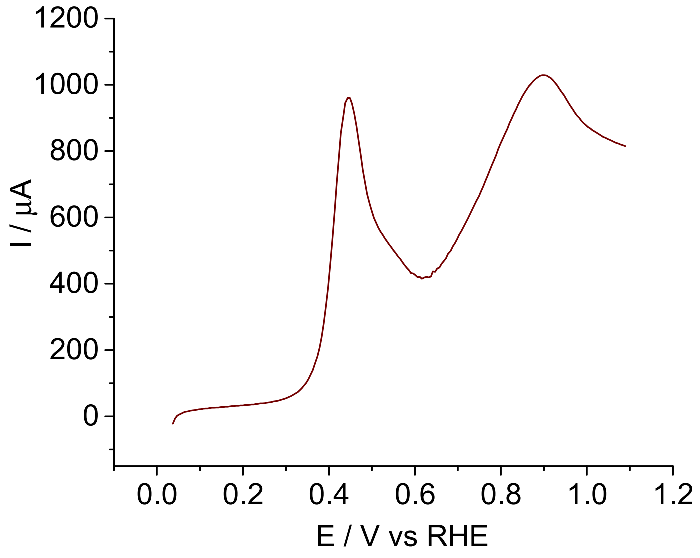

Figure S2. Linear Sweep Voltammogram showing the oxidation of 30 mM AA on a Pt electrode covered with the aniline-piperazine copolymer. AA oxidation peak is centered at 0.89 V.
